# Few-shot disease recognition algorithm based on supervised contrastive learning

**DOI:** 10.3389/fpls.2024.1341831

**Published:** 2024-02-07

**Authors:** Jiawei Mu, Quan Feng, Junqi Yang, Jianhua Zhang, Sen Yang

**Affiliations:** ^1^School of Mechanical and Electrical Engineering, Gansu Agricultural University, Lanzhou, China; ^2^Agricultural Information Institute, Chinese Academy of Agricultural Sciences, Beijing, China; ^3^National Nanfan Research Institute, Chinese Academy of Agricultural Sciences, Sanya, China

**Keywords:** plant disease recognition, few-shot learning, meta-learning, supervised contrastive learning, nearest-centroid classification

## Abstract

Diseases cause crop yield reduction and quality decline, which has a great impact on agricultural production. Plant disease recognition based on computer vision can help farmers quickly and accurately recognize diseases. However, the occurrence of diseases is random and the collection cost is very high. In many cases, the number of disease samples that can be used to train the disease classifier is small. To address this problem, we propose a few-shot disease recognition algorithm that uses supervised contrastive learning. Our algorithm is divided into two phases: supervised contrastive learning and meta-learning. In the first phase, we use a supervised contrastive learning algorithm to train an encoder with strong generalization capabilities using a large number of samples. In the second phase, we treat this encoder as an extractor of plant disease features and adopt the meta-learning training mechanism to accomplish the few-shot disease recognition tasks by training a nearest-centroid classifier based on distance metrics. The experimental results indicate that the proposed method outperforms the other nine popular few-shot learning algorithms as a comparison in the disease recognition accuracy over the public plant disease dataset PlantVillage. In few-shot potato leaf disease recognition tasks in natural scenarios, the accuracy of the model reaches the accuracy of 79.51% with only 30 training images. The experiment also revealed that, in the contrastive learning phase, the combination of different image augmentation operations has a greater impact on model. Furthermore, the introduction of label information in supervised contrastive learning enables our algorithm to still obtain high accuracy in few-shot disease recognition tasks with smaller batch size, thus allowing us to complete the training with less GPU resource compared to traditional contrastive learning.

## 1 Introduction

Plant diseases are a significant factor that impacts crop yield and quality (Flood, 2010). In the traditional method of plant disease identification, the diagnosis is usually made by a human based on plant symptoms (Fang and Ramasamy, 2015), which relies heavily on human experience and is highly subjective. Moreover, this method is time-consuming, inefficient and cannot realize the automation of disease detection. In developing countries, accurate disease recognition is certainly a challenge for farmers with limited education. Misjudging or omitting the type of disease can result in missing the optimal period for disease control, leading to inappropriate use of pesticides, increased risk of crop yield reduction and environmental pollution. Therefore, the creation of an automatic recognition system for plant diseases is very helpful to alleviate the above situations (Shruthi et al., 2019), which would enable farmers to recognize the type of disease in time and take the right control measures to minimize the losses.

Plant disease recognition methods based on computer vision have been widely studied and practical results have been achieved. In recent years, the disease recognition methods based on deep learning have become the mainstream due to its faster detection speed and higher accuracy in comparison with traditional methods. Kawasaki et al. (2015) proposed a novel plant disease detection system based on CNN. Using a total of 800 cucumber leaf images for training, which resulted in an average accuracy of 94.90% on two disease categories and one non-disease category. Mohanty et al. (2016) used 54,306 diseased and healthy plant leaf images from PlantVillage to train a deep convolutional neural network to recognize 14 plants and 38 species, achieving an accuracy of 99.35% on the testing set. Ferentinos (2018) trained multiple model architectures on 25 different plants in the combination of 58 different classes of plants and diseases by means of deep learning, and the best in terms of recognition performance was the VGG network with the accuracy of 99.53%. Picon et al. (2019) used an adaptive algorithm based on deep residual neural networks for the detection of multiple plant diseases in 8178 images with a balanced accuracy of 87%. Liu et al. (2019) used two lightweight networks, MobileNet and Inception V3, to realize the recognition of PlantVillage on Android mobile phones. Barman et al. (2020) used MobileNet and self-structured classifier (SSCNN) to detect and classify leaf diseases in citrus growth stages. Experiments were conducted on 2,939 citrus leaf samples and the results showed that the accuracy of MobileNet and SSCNN was 92% and 99%, respectively. Saleem et al. (2020) trained several CNNs on PlantVillage and concluded that the Xception architecture trained with the Adam optimizer achieved the highest verification accuracy and F1 value, which was 99.81% and 99.78%, respectively. Zhou et al. (2021) proposed a vegetable disease recognition model with complex background based on region proposal and progressive learning (PRP-Net), and the average recognition accuracy reached 98.26% for 6 kinds of disease. Hassan and Maji (2022) proposed a new deep learning model utilizing initial layers and residual connection, achieving 99.39% accuracy on the dataset PlantVillage, 99.66% on the rice disease dataset, and 76.59% on the cassava dataset. Chakraborty et al. (2022) explored some CNN models for automatic recognition of late blight and early blight, with VGG 16 having the highest accuracy of 92.69%. Liu and Zhang (2023) proposed an Inception-V3 based transfer learning method called PiTLiD, which was shown to outperform the comparison method with 99.45% accuracy.

Although deep learning methods have delivered positive outcomes in plant disease recognition work, these methods usually require a large amount of labeled data to learn the differences between disease features or similar diseases. However, the truth is that collecting a large number of images of a particular plant disease is extremely expensive, and some rare diseases are very scarce. In addition, labeling large quantities of disease images is a time-consuming process that requires a large number of professionals, resulting in a high learning cost.

To address the concern that deep learning heavily depends on extensive labeled data, researchers have introduced the concept of Few-shot Learning (FSL) (Ravi and Larochelle, 2016; Wang et al., 2020; Zhao et al., 2020; Jadon and Jadon, 2023), which draws on the ability of humans to acquire knowledge swiftly. Unlike traditional networks, the few-shot learning aims to train learning tasks with fewer samples, that is, to train the classifiers with good performance by inputting only one or a few labeled images. The concept of N-Way K-Shot is often used to describe the specific problems encountered by FSL. In this case, the support set represents the small dataset used in the training phase and generates reference information for the second testing phase. The query set is the task that the model actually needs to predict. Note that the query set classes never appear in the support set. N-way K-shot represents a support set with N categories and K samples per category, then the entire task only has N * K samples. Many strategies can be used in FSL, such as meta-learning, learning fine-tuning, metric learning, and data augmentation. Meta-learning aims to learn optimal initial values of the model from a large number of prior tasks, utilizing previous knowledge to expedite the learning process of the model in new tasks (Vanschoren, 2018). Learning fine-tuning is given a trained basic network, which is obtained by training on a large-scale dataset containing rich labels, and then fine-tune to the domain-specific data. This approach usually gives good results with a little training on a small number of samples. A typical approach is MAML (Finn et al., 2017), which is based on the idea of learning an initialization parameter, and when this initialization parameter encounters new tasks, only a few steps of gradient descent using a small number of samples are required to achieve good results. Metric learning is learning an embedding function that maps the input space to a new feature representation space, where there is a similarity metric to distinguish between the classes. When faced with new tasks, using this representation function to map the classified sample points inside the representation space and using the similarity metric comparison to classify. Classical methods such as Siamese network (Koch et al., 2015), Matching network (Vinyals et al., 2016), Prototypical network (Snell et al., 2017) and Relation network (Sung et al., 2018) are all belong to metric learning. Data augmentation aims at increasing the number of samples when the number of samples is small and improving the generalization ability of the model. Common methods include rotating, flipping, cropping, panning and adding noise to the images.

The work on plant disease recognition have been explored by researchers using FSL. Argüeso et al. (2020) designed a few-shot learning architecture based on Inception v3 network and SVM classifier. Triplet loss was introduced to learn image embeddings, and more refined multi-class SVM was introduced to effectively learn new class boundaries from a few examples. Xiao et al. (2021) compared the disease recognition performance of matching network, prototypical network and relational network on PlantVillage. The average accuracy of the three networks under 5-way 1-shot condition was 72.29%, 72.43% and 69.45%, respectively. The average accuracy of the three networks under the 5-way 5-shot condition was 87.11%, 87.50% and 82.92%, respectively. Liang (2021) employed metric learning method to classify the cotton leaf disease spots. Wang et al. (2021) proposed a few-shot recognition model for vegetable diseases in complex contexts based on image text collaborative representation learning (ITC-Net), which combined the disease image modal information with the disease textual modal information. They utilized the correlation and complementarity between the two types of diseases to achieve the collaborative recognition of disease features. Wang and Wang (2021) suggested an improved meta-learning method (IMAL) for few-shot classification of plant diseases. The result showed that their method was superior to many current few-shot learning methods. Li and Chao (2021) proposed a semi-supervised method for recognizing plant leaf diseases, which was single-shot semi-supervised method and iterative semi-supervised method, respectively. The former achieved the average accuracy of 92.6% at 6-way 10-shot, while the latter achieved the average accuracy of 90% at 6-way 5-shot. Chen L. et al. (2021) presented a few-shot method for detecting plant diseases called LFM-CNAPS, and the result showed that the model could detect unseen plant diseases using only 25 annotated examples with the average accuracy of 93.9%. Lin et al. (2022a) proposed a network based on the meta-baseline few-shot learning method, and combined the cascaded multi-scale features with channel attention. Under the optimal configuration, the accuracy of 5-way 1-shot task and 5-way 5-shot task reached 61.24% and 77.43% respectively in the task of single plant, and 82.52% and 92.83% respectively in the task of multiple plants. Tassis and Krohling (2022) used a dataset composed of biotic stresses in coffee leaves as a case study to evaluate the performance of few-shot learning in classification task and severity estimation task, respectively. Lin et al. (2022b) introduced frequency representation into few-shot learning paradigm for plant disease recognition, designed the discrete cosine transform mode to convert RGB color images to frequency domain, and proposed a learning-based frequency selection method to select information frequency. The data setup for this work simulated two application scenarios: for mixed plant targets, the recognition accuracy of the 5-way 5-shot task could reach 95% when expanding a new class, and for single plant target, the recognition accuracy of the 5-way 5-shot task could reach 80% when expanding a new disease.

So far, FSL has not been sufficiently investigated for plant disease recognition, and new learning paradigms are expected to provide the impetus for improving disease recognition accuracy. Contrastive learning is usually a self-supervised learning method, which pre-trains a model with a large amount of unlabeled data to learn feature representation. The model can be suitable for downstream tasks by acting as a feature extractor and training a classifier/regressor on a labelled dataset. Self-supervised learning uses unlabeled agent tasks on source task data in the hope that generalizable feature representations can be learned from the source tasks for rapid adaptation in the target tasks. Contrastive learning has been widely verified by experiments for its generalization in downstream tasks (Huang et al., 2023), and has shown very high accuracy in visual classification tasks (Caron et al., 2020; Chen et al., 2020b; Grill et al., 2020; Chen X. et al., 2021).

The typical contrastive learning method usually obtains two subsamples from an original image by image augmentation. The pair of subsamples forms a pair of positive pairs and forms negative pairs with the subsamples of other images. The class information of the images is not used within such a setup, so there is no way to know which images belong to the same class, and therefore there is no way to keep the features of similar images close to each other. In order to train a high-performance encoder, a very large batch size or memory bank is usually required, which is very demanding on the GPU hardware (Chen et al., 2020; Chen X. et al., 2021). The supervised contrastive learning proposed by Khosla et al. (2020) allows to use the image class labels, and the basis of training has changed from whether they come from the same image to whether they belong to the same class. Positive samples from multiple other images in the same class may exist for each anchor during loss function computation. This has the advantage of bringing the feature representations of similar samples closer together in hypersphere space. The additional benefit is that the loss function contains richer positive samples in the computation, which helps to reduce the batch size, thus alleviating the hardware requirements. In fact, we implement the training using only 4 GPUs (NVIDIA RTX 3070). Compare to the previous work (e.g., Chen et al., 2020), the requirements for the training hardware configuration are much lower.

Essentially, both FSL and contrastive learning transfer knowledge from a set of source tasks, thereby reducing the need to collect a large amount of labeled training data for the target tasks. Therefore, for few-shot plant disease recognition tasks, we consider combining them in a framework to improve the accuracy under few-shot condition. Based on this intuition, we propose a few-shot disease image classification algorithm based on supervised contrastive learning (Supervised Contrastive Few-shot Learning, SC-FSL). In the pre-training phase, using the label information of the disease to carry out supervised contrastive learning training, so as to obtain an encoding network with strong generalization ability for the disease recognition tasks. In the second phase, the encoder learned in the previous phase is used as a feature extractor for the diseases, by training a nearest-centroid classifier, which carries out the few-shot disease recognition tasks. The experiments demonstrate that the use of feature extractors with good generalization performance, obtained in contrastive learning by learning from a large amount of disease data, has a significant effect on the subsequent improvement of disease recognition accuracy for few shots. The success of the approach indicates that adopting large-scale training in the first phase can enable the feature extractor to achieve better feature clustering performance, which helps to improve the accuracy of the classifier in the second phase of FSL. We believe that this kind of training strategy may provide a new paradigm for FSL. The result of a recent work also supported our idea (Xie et al., 2022). However, our implementation is quite different from this work.

The main contributions of this work are two folds:

1. We introduce the paradigm of contrastive learning into the few-shot learning process. Firstly, an encoder with strong generalization ability is learned by contrastive learning and a large number of disease images in the open plant disease dataset. Secondly, for the specific few-shot task of disease recognition, we use the encoder as a disease feature extractor, and train the disease classifier based on distance metrics and nearest-centroid principle.
2. Thanks to the strategy of supervised contrastive learning, a large number of positive and negative samples can be input simultaneously during one iteration of training the disease encoder, which reduces the size of the batch and allows us to use fewer GPU resources to complete the training.

The rest of this paper is as follows: Section 2 introduces methods and materials, including the overall architecture of SC-FSL, supervised contrastive learning, nearest-centroid classification network, the experimental dataset and evaluation methods. In Section 3, the elaborative experiments are carried out and results are described. This part includes the effects of data augmentation, batch size and iteration times, encoding networks and temperature coefficients on the performance of the model, as well as the performance comparison with several classical FSL algorithms, and the application in potato disease of natural scenes. Section 4 summarizes this work.

## 2 Materials and methods

### 2.1 Architecture of SC-FSL

**Figure 1:**
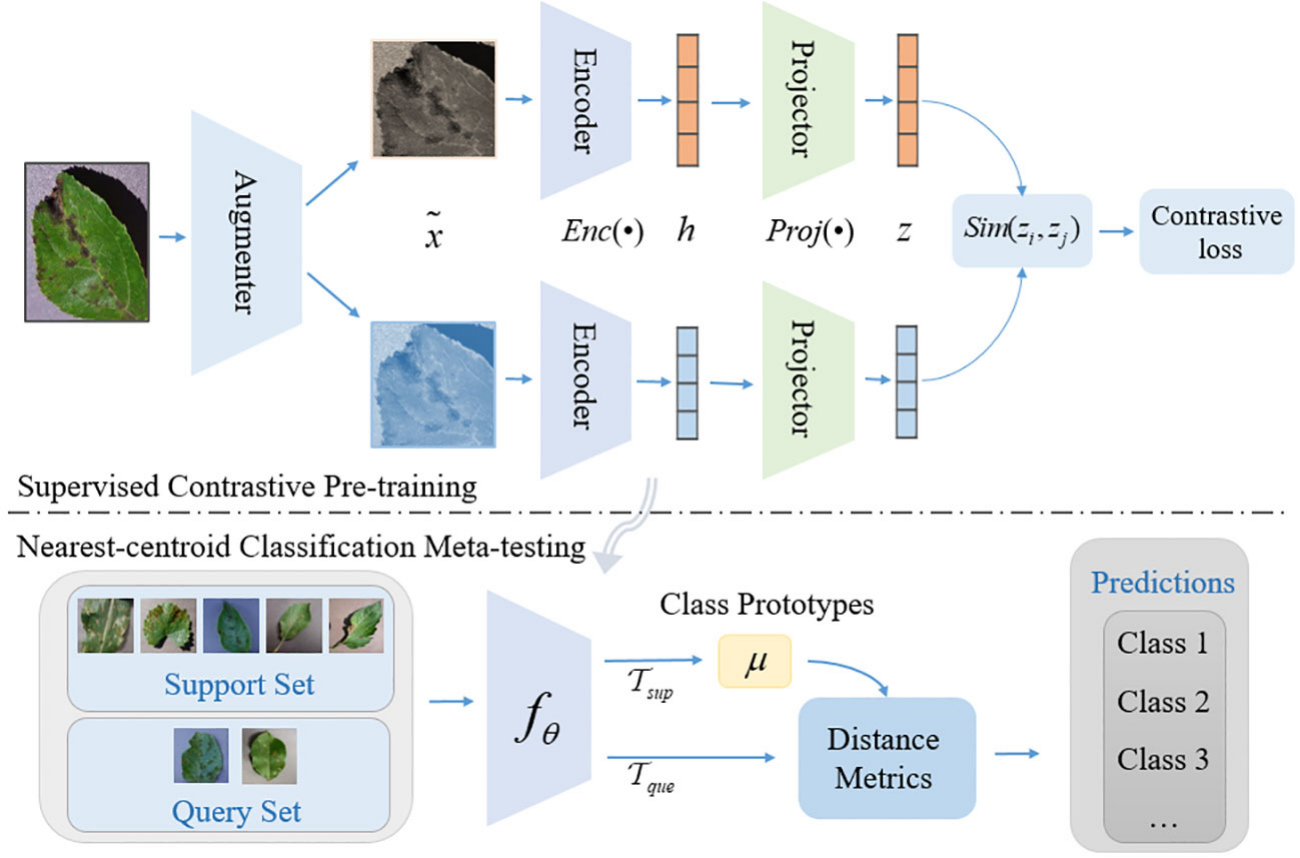
Overview of supervised contrastive few-shot learning framework.

The overall architecture of SC-FSL is shown in **Figure 1**. The method is divided into two phases. In the first phase, the supervised contrastive learning is employed to pre-train the encoding network to obtain more generalized representation. In the second phase, the pre-trained encoding network is frozen and treated as the feature extractor of plant disease. We train a nearest-centroid classification network on the few-shot dataset to complete the final disease classification prediction.

The modules in the **Figure 1** are introduced as follows:

1. Data Augmentation, denoted as: *Aug*(•), for each input sample *x*, two random data augmentations $\tilde{x_{i}}$ and $\tilde{x_{j}}$ are generated by *Aug*(•) and regarded as a pair of positive samples, each of which represents a different view of the data.
2. Encoder Network, denoted as: *Enc*(•), the two enhanced samples are input into the same encoding network to obtain a pair of vector representations $h_{i}=Enc\left(\tilde{x_{i}}\right)$ and $h_{j}=Enc\left(\tilde{x_{j}}\right)$ . Then the representation is normalized, which can improve the generalization ability to a certain extent. The encoding network can be any convolutional neural network. We use ResNet in this study, which is also the *f_θ_* of the lower part of **Figure 1** as a few-shot feature extractor.
3. Projection Network, denoted as *Proj*(•), maps the representation vector obtained after the encoding network to the contrastive loss space. Usually, *Proj*(•) is instantiated as a Multi-Layer Perceptron (MLP) and discarded after the contrastive training (Tian et al., 2020).
4. Contrastive loss, denoted as ℒ , aims to decrease the distance between positive samples in the representation space, and push the distance between the negative samples, so as to achieve the representation learning.

### 2.2 Supervised contrastive learning

The idea of supervised contrastive learning is that image label information is used in the embedding space so that representations from the same category in the feature representation space are closer together than representations from different categories. This method extends self-supervised contrastive learning to a fully supervised training paradigm. For plant disease recognition tasks, this allows us to effectively use disease label information. The idea behind the method is to gather the disease sample clusters with the same class labels as much as possible in the feature representation space, while the disease sample clusters with different class labels are as far away as possible, so as to improve the discriminant ability of the model in the case of intra-class diversity and inter-class similarity.

**Figure 2:**
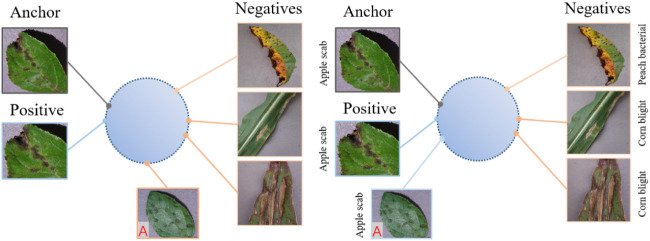
Contrastive loss in the embedding space.

**Figure 2** shows an illustration of self-supervised contrastive learning and supervised contrastive learning in the feature representation space. In self-supervised contrastive learning, the positive samples of each anchor are obtained by their own data augmentation, which are closer to the anchor in the feature representation space. The negative samples are composed of all other samples remaining in the batch and should be kept as far away from the anchor as possible. In supervised contrastive learning, since the label information of each sample is known, all samples belonging to the same class are regarded as positive samples, and the samples of other classes are regarded as negative samples. In **Figure 2**, Sample A (marked in red) belongs to the same class as the anchor, but it is not acquired through anchor augmentation. Thus, A is pushed further away from the anchor in self-supervised contrastive learning. However, in supervised contrastive learning, the use of class label information can narrow the distance between it and the anchor, so that the diseases belonging to the same class are arranged more closely in the feature representation space.

To compute the supervised contrastive loss, in addition to considering the negative example samples, the positive example samples are considered for each anchor. This is different from the traditional self-supervised contrastive learning. In the traditional way, only single positive sample can be seen, and the rest are all negative samples. Meanwhile, the positive samples in supervised contrastive learning are sampled from other instances with the same anchor class. The supervised contrastive loss function is designed as follows (Equations 1–4):


(1) $${ℒ}^{\sup}=\sum_{i=1}^{2N}{ℒ}_{i}^{sup}$$

(2) $${ℒ}_{i}^{sup}=\frac{1}{2N_{y_{i}}-1}\sum_{j=1}^{2N}{\text{𝟙}}_{\left[i\neq j\right]}\cdot {\text{𝟙}}_{\left[y_{i}=y_{j}\right]}\cdot \ell_{ij}$$

(3) $$\ell_{ij}=-log\frac{exp\left(sim(z_{i},z_{j\left(i\right)}\right)/\tau )}{\sum_{k=1}^{2N}{\text{𝟙}}_{\left[i\neq k\right]}exp\left(sim(z_{i},z_{k}\right)/\tau )}$$

(4) $$\text{sim}\left({\text{z}}_{i},z_{j\left(i\right)}\right)=z_{i}^{T}z_{j\left(i\right)}/\left(∥z_{i}∥∥z_{j\left(i\right)}∥\right)$$

The notation of the above equation is explained as follows. *i* denotes the anchor. $N_{y_{i}}$ represents the total number of images with the same label *y_i_* as *i*. $z_{i}=Proj\left(Enc\left({\tilde{x}}_{i}\right)\right)$ represents the output of the feature ${\tilde{x}}_{i}$ after it has been encoded by the encoding network *Enc*(•) and projected by the projection network *Proj*(•). $1_{\left[i\neq k\right]}\in \left\{0,1\right\}$ is an indicator function, if and only if i≠k is 1, otherwise it is 0. *j*(*i*) denotes the positive example samples of anchor *i*, and the other 2(N-1) terms denote the negative example samples. *τ* denotes the scalar temperature coefficient. $sim(z_{i},z_{j\left(i\right)})$ denotes the similarity measure function between *z_i_* and *z_j_*_(_*_i_*_)_.

**Algorithm 1** gives the pseudo-code of the proposed method. **Table 1** lists the symbols used in **Algorithm 1** and their meanings.

**Table 1:** Symbols and meaning.

Symbols	Meaning
*D*	training dataset
*N*	batch size
*τ*	temperature
*Enc*	encoder network
*Proj*	projection network
${\left\{x_{k}\right\}}_{k=1}^{N}$	minibatch in training dataset
*t*	data augmentation operator
*t'*	data augmentation operator

Algorithm 1 **Supervised contrastive Pre-training’s main learning algorithm.**

**1  input:** *D*, batch size *N*, *τ*, *Enc*, *Proj*.
**2  for** sampled minibatch 
${\left\{x_{k}\right\}}_{k=1}^{N}$
~*D* **do**
**3   for all** 
k∈{1,…,N}
 **do**
**4    ** 
${\tilde{x}}_{2k-1}=t\left(x_{k}\right)$
                 # the first augmentation
**5    ** 
$h_{2k-1}=Enc\left({\tilde{x}}_{2k-1}\right)$
                        # representation
**6    ** 
$z_{2k-1}=Proj\left(h_{2k-1}\right)$
                             # projection
**7    ** 
${\tilde{x}}_{2k}=t^{\prime }\left(x_{k}\right)$
                 # the second augmentation
**8    ** 
$h_{2k}=Enc\left({\tilde{x}}_{2k}\right)$
                             # representation
**9    ** 
$z_{2k}=Proj\left(h_{2k}\right)$
                                   # projection
**10   end for**
**11   for all** 
i∈{1,…,2N}
 and 
j∈{1,…,2N}
 do
**12  ** 
$sim\left(z_{i},z_{j\left(i\right)}\right)=z_{i}^{\text{T}}z_{j\left(i\right)}/\left(∥z_{i}∥∥z_{j\left(i\right)}∥\right)$
 # pairwise similarity
**13   end for**
**14   ** 
$\ell \left(i,j\right)=-log\frac{exp\left(sim(z_{i},z_{j\left(i\right)}\right)/\tau )}{\sum_{k=1}^{2N}1_{\left[i\neq k\right]}exp\left(sim(z_{i},z_{k}\right)/\tau )}$

**15   ** 
${ℒ}_{i}^{sup}=\frac{1}{2N_{y_{i}}-1}\sum_{j=1}^{2N}1_{\left[i\neq j\right]}\cdot 1_{\left[y_{i}=y_{j}\right]}\cdot \ell_{ij}$

**16   ** 
${ℒ}^{sup}=\sum_{i=1}^{2N}{ℒ}_{i}^{sup}$

**17   ** update networks *Enc* and *Proj* to minimize 
${ℒ}^{sup}$

**18  end for**
**19  return** encoder network *Enc*(•)

The output of supervised contrastive learning is not a classifier, but a superior encoder which brings similar classes of data as close together as possible in feature space and excludes different classes of data. The encoder can be used as a feature extractor in downstream classification tasks, depending on the specific objects recognized and the number of categories. When implementing a specific classification task, it can be trained again after simply adding a classification layer behind the feature extractor. Any CNN network can used as the encoder. In fact, we use the ResNet in the later experiments, deleted its fully connected layer (classification layer) and only retained the previous convolutional layer and residual layer.

It is worth noting that the paradigm of contrastive learning belongs to large-scale learning. Therefore, in the first stage of SC-FSL we still adopt the strategy of large-scale training. Only in the second stage, the M-way N-shot method is used. This is different from traditional FSL in which M-way N-shot method is usually employed in both stages.

### 2.3 Nearest-centroid classification network

The few-shot disease recognition model based on supervised contrastive learning is a two-phase few-shot learning method. In the first phase, an encoding network for learning the feature representation of disease images is trained by supervised contrastive learning method. In the second phase, the trained encoding network is used as the feature extractor, and a nearest-centroid classification network is trained by using the few-shot general learning paradigm (N-way K-shot), so as to realize the few-shot disease recognition tasks. Note: in this stage, the feature extractor is frozen and its parameters are not modified in any way. We simply train the parameters of the classifier.

**Figure 3:**
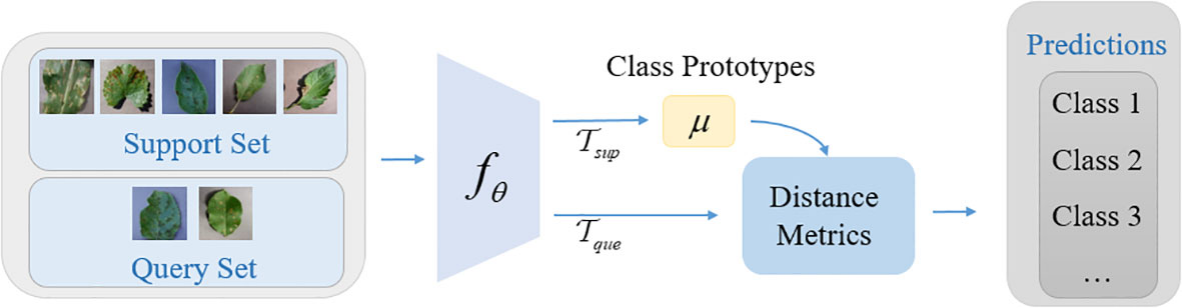
The nearest-centroid classification network structure diagram.

The basic idea of the nearest-centroid classification network is to create a prototype representation for each class, which is called class prototype. For the query sample that need to be classified, the distance metric between the query sample feature vector and the class prototype is calculated, and the corresponding class of the class prototype with the smallest distance is selected as the predicted class. The structure of the nearest-centroid classification network is shown in **Figure 3**.

Firstly, the sample of training set *x_i_* of each class of disease images is mapped to the feature representation by nonlinear feature encoder *f_θ_*, and then the mean value of each class in the feature representation is calculated separately as the class prototype $\mu_{m}\left(m=1\sim M\right)$ (M is the number of classes), and the calculation formula is shown in Equation 5:


(5) $$\mu_{m}=\frac{1}{\left|S_{m}\right|}\sum_{(x_{i},y_{i})\in S_{m}}f_{\theta }\left(x_{i}\right)$$

where *μ_m_* denotes the class prototype of the class m. *f_θ_* denotes the nonlinear feature mapping function. *S_m_* denotes the set of data samples with the class m in the training set S, and |*S_m_* | is the total number of samples. (*x_i_*, *y_i_*) denotes the sample and the corresponding label of training set.

The Euclidean metric is used as the metric function between the query set samples and the class prototypes, then the class distribution for a given sample *x* is (Equations 6, 7):


(6) $$p\left(y=m|x\right)=\frac{exp\left(-d\left(f_{\theta }\left(x\right),\mu_{m}\right)\right)}{\sum_{m^{\prime }}exp\left(-d\left(f_{\theta }\left(x\right),\mu_{m^{\prime }}\right)\right)}$$

(7) $$d\left(f_{\theta }\left(x\right),\mu_{m}\right)=\sqrt{{\left(f_{\theta }\left(x\right)-\mu_{m}\right)}^{2}}$$

where *μ_m’_* denotes the class prototype of each class in *μ_m_.*

### 2.4 Dataset

**Figure 4:**
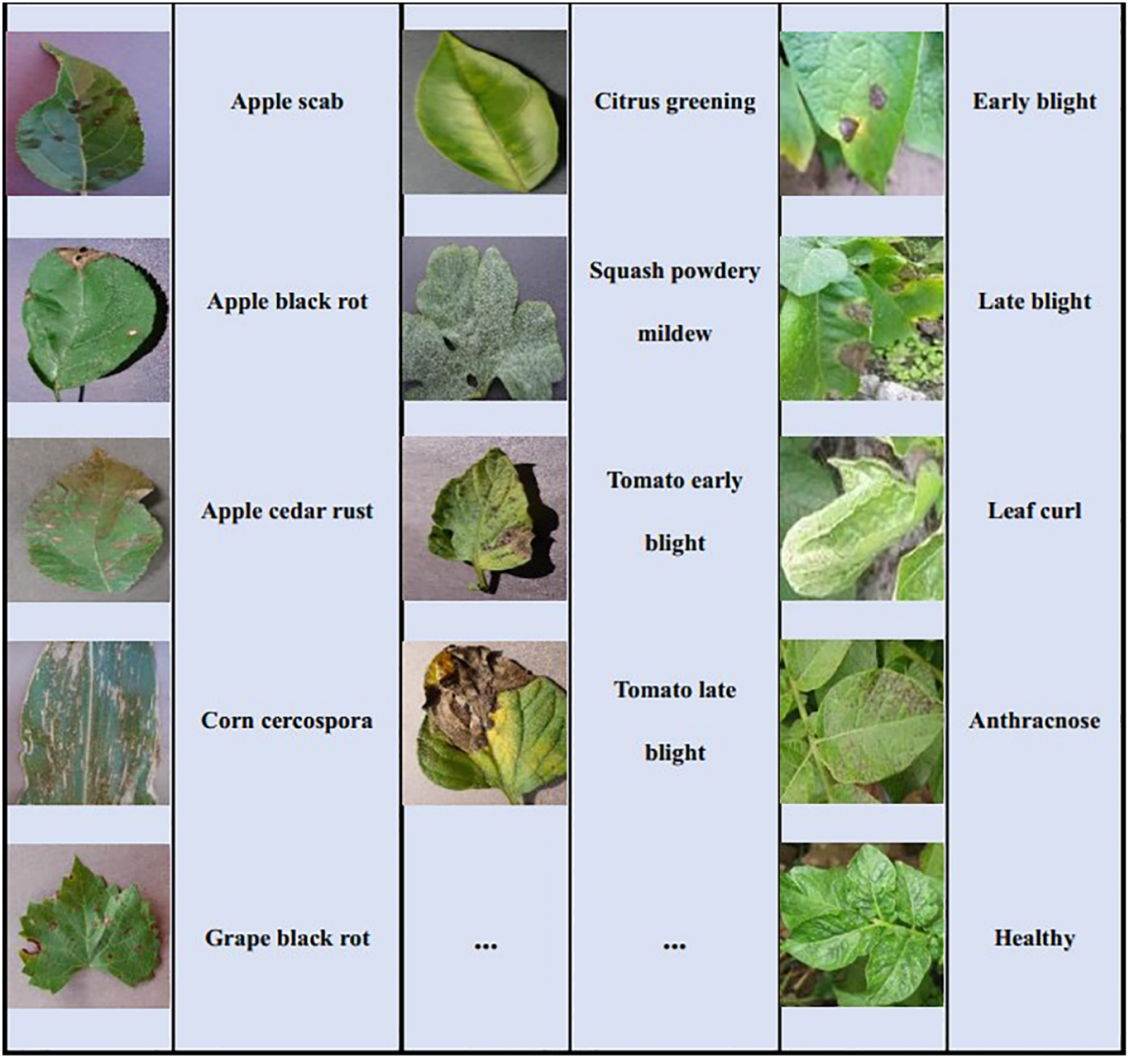
Some examples of disease samples from PlantVillage and PDD.

Two plant disease datasets are utilized in the study to conduct the experiments for few-shot disease recognition. The first is PlantVillage (Hughes and Salathe, 2016) which is the most widely used open-access plant disease image database. It collects 54,306 images of plant disease leaves, including 14 kinds of plants and a total of 38 classes. All images in this dataset are taken under laboratory conditions with controlled lighting and simple backgrounds. **Table 2** displays all the plants and their disease types, and some examples in PlantVillage are given in **Figure 4A**. The size of each image is 256*256*3.

**Table 2:** Information of PlantVillage.

Class	Plant name	Disease	Images	Class	Plant name	Disease	Images
**1**	Apple	Scab	630	**20**	Pepper	Healthy	1478
**2**		Black rot	622	**21**	Potato	Early blight	1000
**3**		Cedar rust	275	**22**		Healthy	152
**4**		Healthy	1645	**23**		Late blight	1000
**5**	Blueberry	Healthy	1502	**24**	Raspberry	Healthy	371
**6**	Cherry	Healthy	854	**25**	Soybean	Healthy	5090
**7**		Powdery mildew	1052	**26**	Squash	Powdery mildew	1835
**8**	Corn	Cercospora	513	**27**	Strawberry	Healthy	456
**9**		Rust	1192	**28**		Leaf scorch	1109
**10**		Healthy	1162	**29**	Tomato	Bacterial spot	2127
**11**		Northern leaf blight	985	**30**		Early blight	1000
**12**	Grape	Black rot	1180	**31**		Healthy	1591
**13**		Black measles	1383	**32**		Late blight	1909
**14**		Healthy	423	**33**		Leaf mold	952
**15**		Isariopsis leaf spot	1076	**34**		Septoria leaf spot	1771
**16**	Orange	Citrus greening	5507	**35**		Spider mites	1676
**17**	Peach	Bacterial spot	2297	**36**		Target spot	1404
**18**		Healthy	360	**37**		Mosaic virus	373
**19**	Pepper	Bacterial spot	997	**38**		Yellow leaf curl	5357

The second dataset is the Potato Disease Dataset (PDD) in natural scenes. The images of PDD are collected from the Internet by us. Unlike PlantVillage, they are taken under natural light conditions and the background is complex. PDD includes five types of images of potato leaf diseases, namely early blight, late blight, leaf curl, anthracnose and healthy leaves, with 40 images in each class. Some examples of these five diseases are given in **Figure 4B**. To be consistent with the PlantVillage, all images are resized to 256*256*3.

In order to evaluate the transferability of the few-shot disease recognition algorithm proposed in this paper between the source domain and target domain, two scenarios are arranged in the experiments. Scenario A: both source domain data and target domain data are from PlantVillage. Scenario B: The source domain data comes from PlantVillage, while the target domain data comes from PDD. In Scenario A, PlantVillage is split into two non-overlapping parts, one is the source domain and the other is the target domain. We evaluate several factors that affect the performance of SC-FSL, and compare the recognition accuracy of SC-FSL with other nine algorithms. Although the target domain and source domain have different disease classes, the images in them are taken under the same conditions. Therefore, the feature distributions of the two domains are similar. We further evaluate the transferability of our algorithm between two domains with completely different feature distributions in Scenario B. In Scenario A, we randomly select N classes from the source domain or target domain of each task, with K samples per class, and then the entire task has only N × K samples, with K generally not exceeding 20. In Scenario B, each task is constructed in a similar way to Scenario A.

### 2.5 Evaluation methodology

In order to evaluate the performance of SC-FSL, we use top-1 accuracy as the evaluation index. Top-1 accuracy is the most commonly used performance evaluation index for image recognition models. The plant disease recognition in this study is a single-label multi-classification problem, and in the experimental design of few-shot learning, the number of samples in each class is consistent, so the number of each class is well balanced. Therefore, top-1 accuracy is a good overall evaluation criterion. The formula is shown in Equation 8:


(8) $$\varphi =\frac{1}{m}\sum_{i=1}^{m}\text{𝟙}\left(f\left(x_{i}\right)=y_{i}\right)$$

Where 𝟙(·) is output is 1 (condition is true) or 0 (condition is false). *m* is the number of query set samples in a task. *f*(*x_i_*) is the predicted value of the query set sample *x_i_*, and *y_i_* is the true label of *x_i_*. *φ* denotes top-1 accuracy.

In order to describe the uncertainty of accuracy in repeated experiments, we use standard deviation to measure it. Generally, the Bessel formula is used to estimate the standard deviation as shown in Equation 9:


(9) $$s=\sqrt{\frac{\sum_{i=1}^{n}{\left(\varphi -\bar{\varphi }\right)}^{2}}{n-1}}$$

where *s* denotes the standard deviation. *n* denotes the number of repetitions. $\bar{\varphi }$ denotes the average value of top-1 accuracy.

Finally, the final experimental results are evaluated using criteria consistent with classical few-shot learning algorithms such as MAML (Finn et al., 2017), and the style of the results in all the tables in this paper is shown as Equation 10:


(10) $$\bar{\varphi }\pm 1.96\;*\;s/\sqrt{n}$$

The second half of the formula is a measure of the 95% confidence interval. Unless otherwise stated, the accuracy mentioned later in this paper refers to $\bar{\varphi }$ .

## 3 Results and discussion

### 3.1 Experimental setup

The experiment is completed by using the IW4211-8G (SITONHOLY) rack server. The hardware configuration is as follows: Intel^®^ Xeon(R) Gold 6240 CPU @2.60GHz * 72, GPUs are NVIDIA GeForce RTX 3070 * 4, and running memory is 128GB. The software platform is Ubuntu 18.04.6 LTS 64-bit operating system, CUDA Toolkit 11.3 and CUDNN v8.2.0. The programming language is Python 3.7, and the deep learning framework is Pytorch 1.10.2.

The main training parameters of the study are as follows: in the first phase of contrastive learning, SGD with momentum parameter of 0.9 and weight decay rate of 5×10^-4^ is used as the optimizer, and the Warming up strategy is used for warm-up training. Then the Cosine annealing algorithm is used for three times of decay. The initial learning rate is 0.05 and the decay rate is 0.1. All models are trained with 500 epochs. In the second phase, following the N-way K-shot sampling strategy in few-shot classification task, the average accuracy of 600 episodes and their 95% confidence intervals are evaluated and reported.

### 3.2 Impact of different combinations of data augmentation

In contrastive learning, the model constructs positive sample pairs and negative sample pairs by data augmentation. Therefore, we investigate the impact that different data augmentation methods have on recognition accuracy. The benchmark network chosen in the experiment is ResNet 18. It is worth noting that, unless otherwise specified, the encoding network used in the next experiments is ResNet 18, and dataset configuration is Scenario A described in Section 2.4, that is, the source and target dataset are both PlantVillage. For simplicity, we denote A, B, C and D to represent 4 kinds of data augmentations. A is the random length-width ratio cropping, and the random cropping area ratio is 0.2-1.0. B denotes the random horizontal flipping of the images according to the probability, and the flipping probability is 0.5. C is the image color distortion operation that modifies brightness, contrast, and saturation, and for which a probability of 0.8 is applied. D denotes the random conversion of images to grayscale images. **Table 3** shows the evaluation results of few-shot recognition under the conditions of different combinations of data augmentations.

**Table 3:** Effect of data augmentations of SC-FSL on accuracy of plant disease recognition.

Data Aug	Accuracy (%)		Data Aug	Accuracy (%)	
	5-way 1-shot	5-way 5-shot		5-way 1-shot	5-way 5-shot
A	64.16 ± 0.86	79.55 ± 0.65	B+C	67.53 ± 0.87	81.28 ± 0.61
B	54.19 ± 0.85	69.07 ± 0.81	B+D	46.89 ± 0.86	62.49 ± 0.77
C	65.43 ± 0.87	82.65 ± 0.60	C+D	56.54 ± 0.92	73.88 ± 0.70
D	45.35 ± 0.86	57.88 ± 0.88	A+B+C	**78.55 ± 0.81**	**92.90 ± 0.47**
A+B	62.26 ± 0.87	78.01 ± 0.64	A+B+D	49.47 ± 0.92	61.72 ± 0.79
A+C	75.36 ± 0.80	91.55 ± 0.46	B+C+D	57.11 ± 0.90	74.87 ± 0.75
A+D	48.46 ± 0.89	59.39 ± 0.84	A+B+C+D	77.78 ± 0.81	91.78 ± 0.45

Underline indicates the highest recognition accuracy in a single data augmentation; double underline indicates the highest recognition accuracy in a combination of two kinds of data augmentations; bold indicates the highest recognition accuracy in a combination of three kinds of data augmentations.

As can be seen from **Table 3**, for single data augmentation, the highest recognition accuracy is obtained using random color distortion with the probability of 0.8, followed by image random length-width ratio cropping operation. In the case of the combination of two kinds of data augmentations, the combination of random length-width ratio cropping and random color distortion achieves the highest accuracy. As for the combination of three kinds of data augmentations, the highest recognition accuracy is obtained by the combination of random length-width ratio cropping, random horizontal flipping and random color distortion. It can be observed that it seems that the highest accuracy increases as the number of data augmentations increases. However, inappropriate combinations of data augmentation can also reduce the accuracy of disease recognition. For example, if random grayscale appears in a given combination. In fact, when all four data augmentations are present, the accuracy of disease recognition decreases instead. As can be seen from **Table 3**, the accuracy of random grayscale is also the lowest for all scenarios.

The above results show that for the appropriate combination of data augmentations may make the model learn better feature representations, but for plant disease recognition, random grayscale plays a negative role in various combinations. This operation causes the disease image to lose its color. Therefore, it is reasonable to speculate that in the supervised contrastive learning, the color information of the disease enables the encoding network to pull samples of other categories further apart. If the color information is lost, the encoding network cannot effectively cluster the samples of the same disease.

### 3.3 Impact of batch size and epoch

**Figure 5:**
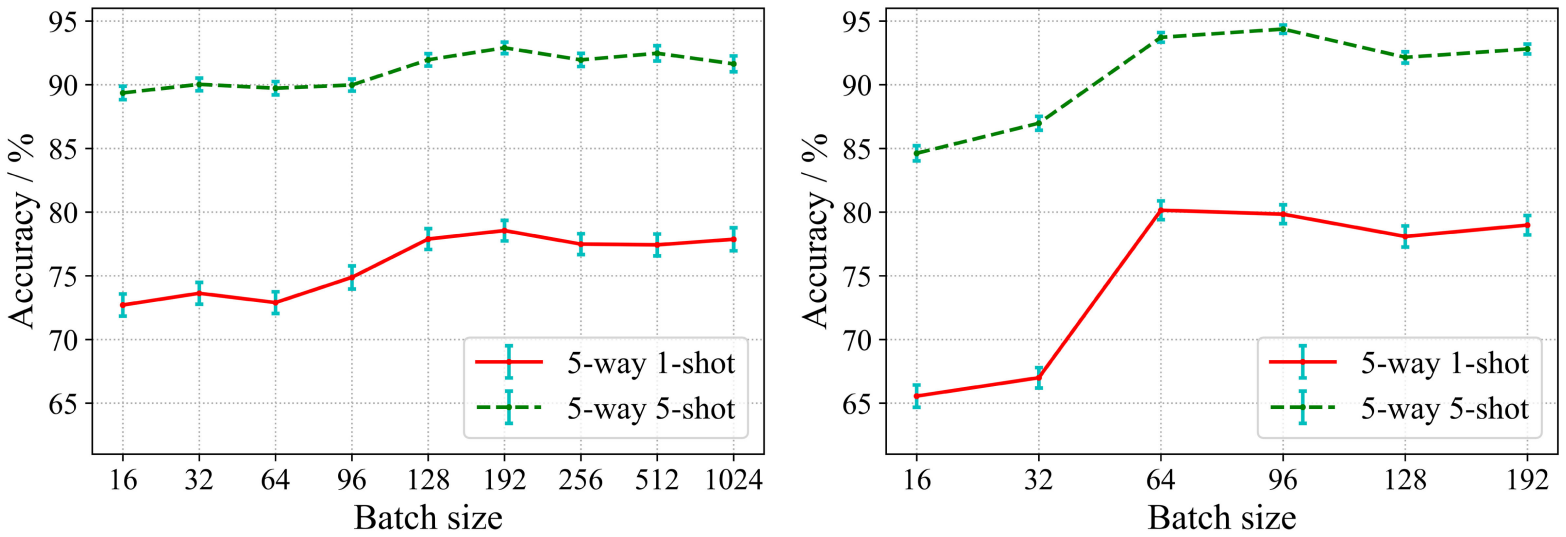
Effect of batch size on accuracy.

In the proposed algorithm, we implement the training of encoding network by supervised contrastive learning in the first phase. The traditional contrastive learning methods suggest larger batch size and more epochs during model training to ensure the generalization performance of the encoding network. However, large batch size and more epochs are extremely demanding on computational resources. In contrast, the supervised training paradigm can be performed in smaller batch size settings. At the same time, the encoding network learned in the first phase directly affects the generalization performance of the classifier in the second phase. Therefore, we study the effects of different batch sizes and epochs on the performance of the model. Two encoding networks with different depths are used in our experiments. One is ResNet 18 which is a shallow residual convolutional neural network, and the other is ResNet 50 which is a deeper residual convolutional neural network. As in Section 3.2, the database configuration for this experiment is also Scenario A. **Figure 5** shows the impact of different batch size settings on the model performance.

As can be seen in **Figure 5A**, at the first, the accuracy of the model gradually increases with respect to the batch size. However, when the batch size exceeds 192, the recognition accuracy tends to be saturated, and the accuracy even decreases slightly when the batch size continues to increase. It can be seen from **Figure 5B** that for ResNet 50, it shows the same trend as ResNet 18. For the deeper encoding network, the model can achieve the highest recognition accuracy when a smaller batch size (64-96). Obviously, the more samples in the support set, the higher the recognition accuracy. Therefore, the accuracy of 5-way 5-shot is higher than 5-way 1-shot for both cases.

The above results exhibit that SC-FSL can bring good performance with smaller batch size. This is in contrast to unsupervised contrastive learning which requires a large batch size (Chen et al., 2020a; He et al., 2020). The results establish that supervised training with label information enables the contrastive learning to perform efficient knowledge transfer for few-shot disease recognition tasks without a large batch size, which greatly reduces the computational requirements.

**Figure 6:**
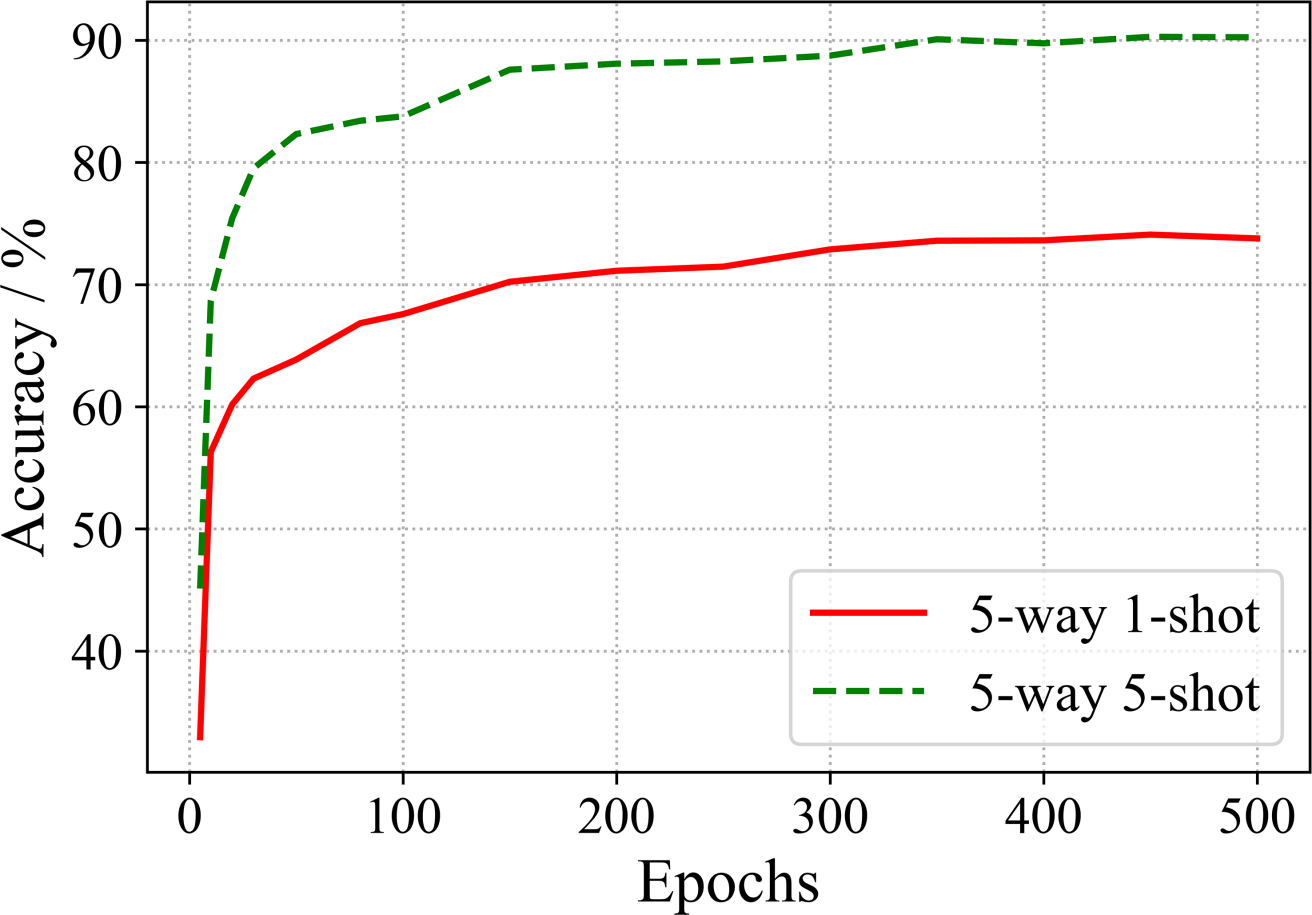
Training epochs vs. accuracy.

In order to observe the effect of training epochs on the performance, we conduct the few-shot recognition evaluation of the model for various epochs. The results of ResNet 18 are shown in **Figure 6**.

From **Figure 6**, one can observe that the recognition accuracy increases with the increase of training epochs. When 350 epochs are trained, the accuracy tends to be saturated.

### 3.4 Impact of temperature coefficient

**Figure 7:**
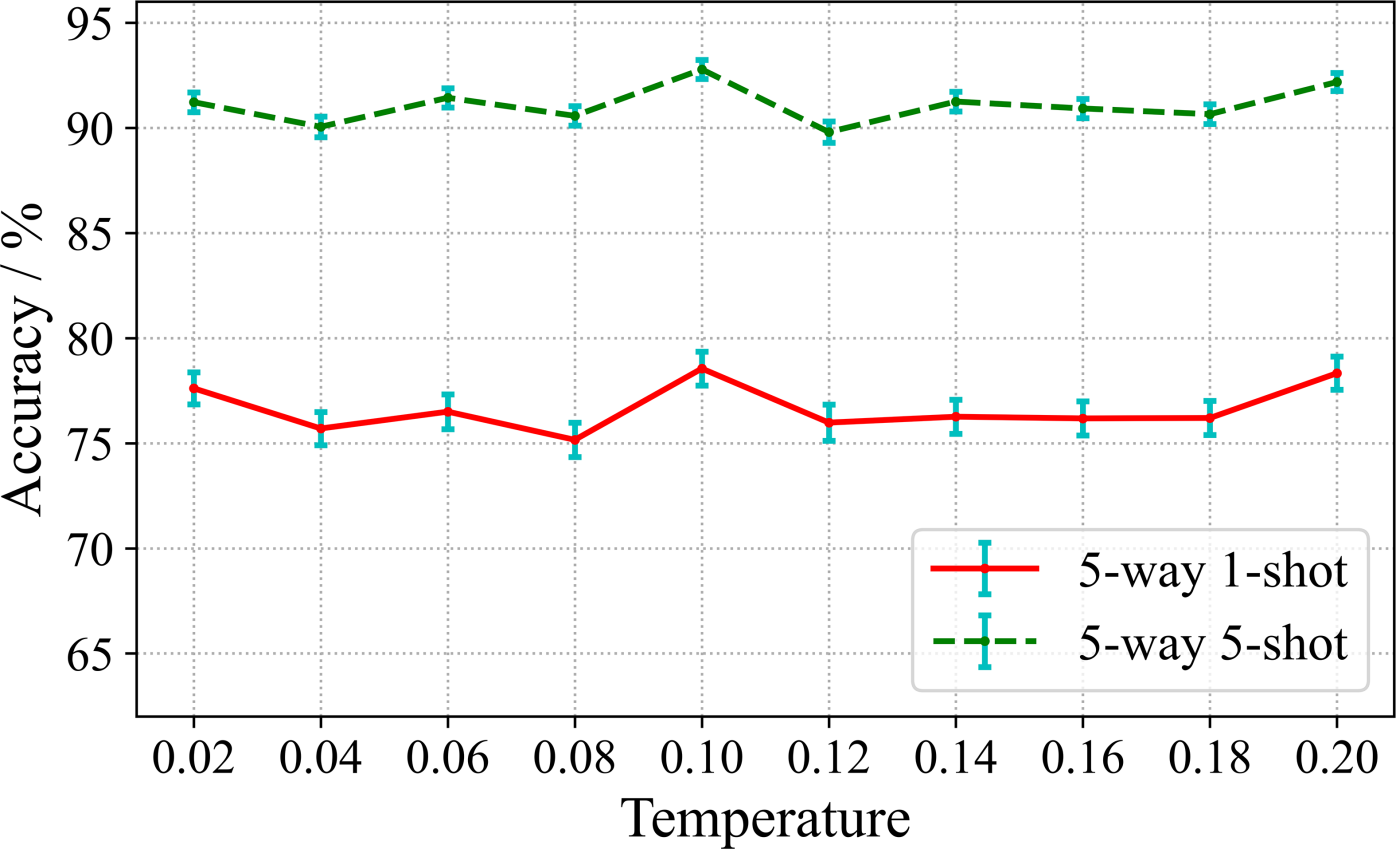
Effect of temperature parameters on accuracy.

According to the Equation 3 of the loss function, the temperature coefficient τ is a hyperparameter used to adjust the contrastive representation learning. Therefore, we study the effect of τ on the performance of few-shot diseases classification. In the experiment, we set the value range from 0.02 to 0.2. The experimental results are shown in **Figure 7**.

**Figure 8:**
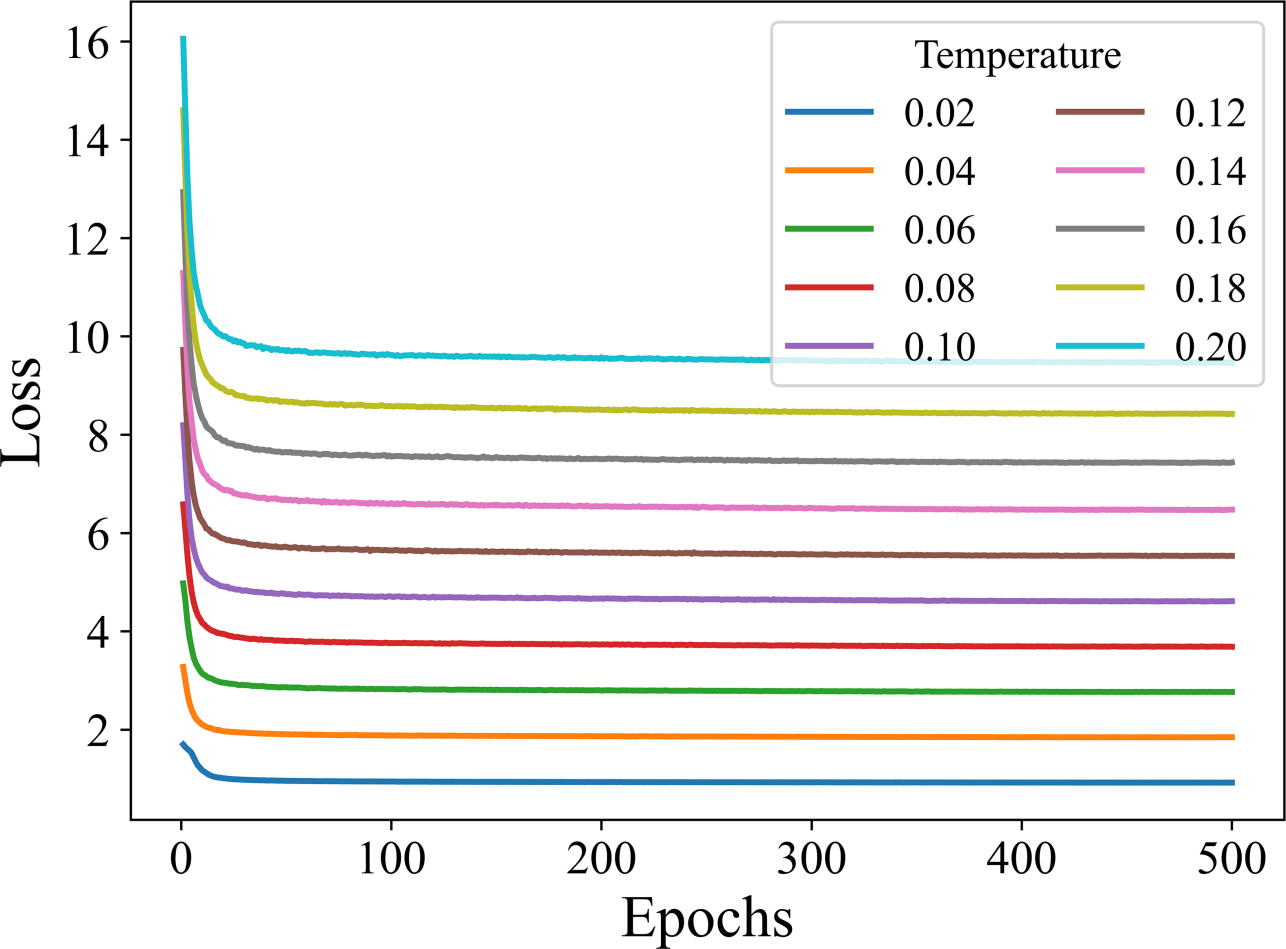
Training loss curve with respect to τ.

It can be seen from **Figure 7**, there are slight fluctuations in the recognition accuracy over the range of τ involved in the experiment. This indicates that the learning effect of the encoding network is relatively stable and the training algorithm is robust over the considered range of τ. The highest recognition accuracy is obtained when τ is set to 0.1 for both 5-way 1-shot and 5-way 5-shot. Furthermore, in order to observe the convergence of the model under different temperature coefficients, we plot the loss curves for 500 epochs of model training under different τ, as shown in **Figure 8**.

As can be seen from **Figure 8**, the supervised contrastive training losses converge well at different τ. Also, the smaller the τ, the smaller the final convergence value of the loss.

### 3.5 Comparison with common FSL algorithms

We compare the disease recognition accuracy of SC-FSL algorithm with nine popular FSL algorithms, including ProtoNet (Snell et al., 2017), MatchingNet (Vinyals et al., 2016), RelationNet (Sung et al., 2018), NegMargin (Liu et al., 2020), MetaBaseline (Chen Y. et al., 2021), MAML (Finn et al., 2017), FEAT (Ye et al., 2020), MELR (Fei et al., 2020) and DeepEMD (Zhang et al., 2020). The various hyperparameters of the proposed method are set to the best value resulted from the aforementioned sections. ResNet 18 is used as a feature extractor in SC-FSL. The few-shot dataset is constructed following the Scenario A. **Table 4** gives the experimental results.

**Table 4:** SC-FSL vs. some state-of-the-art FSL.

Algorithms	Accuracy (%)	
	5-way 1-shot	5-way 5-shot
ProtoNet	75.32 ± 0.80	89.70 ± 0.51
MatchingNet	76.80 ± 0.81	87.85 ± 0.56
RelationNet	74.71 ± 0.83	88.90 ± 0.40
NegMargin	72.40 ± 0.80	90.78 ± 0.47
MetaBaseline	70.07 ± 0.81	87.02 ± 0.51
MAML	69.97 ± 0.96	86.04 ± 0.56
FEAT	74.23 ± 0.03	88.01 ± 0.03
MELR	74.90 ± 0.75	89.02 ± 0.13
DeepEMD	73.87 ± 0.07	88.56 ± 0.06
SC-FSL(ours)	**78.55 ± 0.81**	**92.90 ± 0.47**

The boldface is the best result and the underline the second-ranked result.

It can be seen from **Table 4** that among all the algorithms, the SC-FSL achieves the highest recognition accuracy in both 5-way 1-shot and 5-way 5-shot. The recognition accuracy of 5-way 1-shot is 1.75% higher than that of the second-ranked MatchingNet algorithm, and the accuracy of 5-way 5-shot is 2.12% higher than that of the second-ranked NegMargin. The above results show that SC-FSL is a highly competitive method for FSL.

### 3.6 Impact of different encoding networks

**Figure 9:**
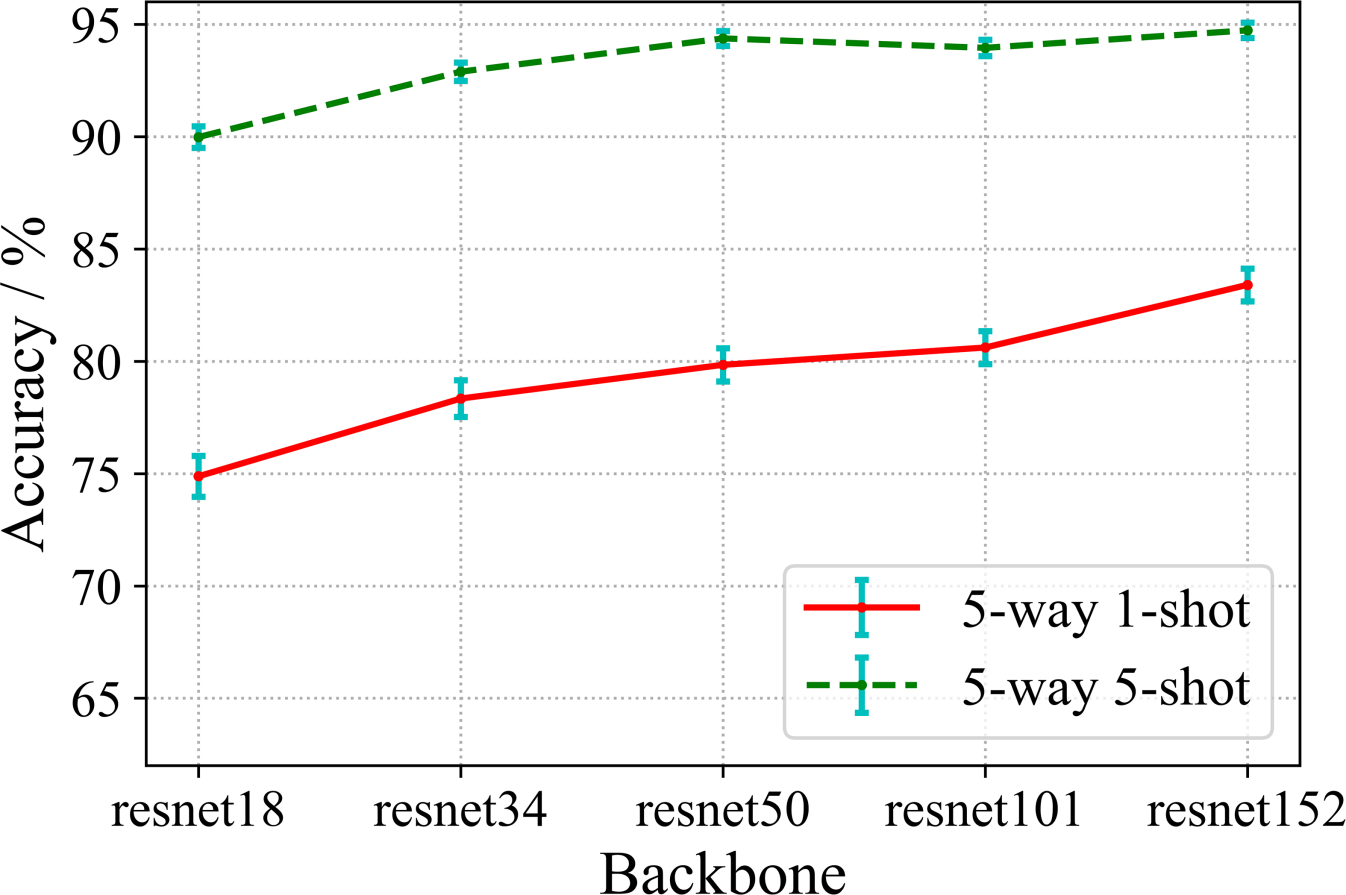
Effect of different encoding networks on accuracy.

The benchmark encoding network selected in the above experiments basically is ResNet 18 which is a relatively shallow residual convolutional neural network. In this section, we will investigate whether the deeper encoding network learn better feature representation by supervised contrastive learning. We implement the training of the shallower encoding networks ResNet 18 and ResNet 34, as well as the deep encoding networks ResNet 50, ResNet 101 and ResNet 152 under the supervised contrastive learning framework, and conduct few-shot disease recognition evaluation. The experimental results are shown in **Figure 9**.

From **Figure 9**, we observe that the recognition accuracies of both 5-way 1-shot and 5-way 5-shot increase with respect to the depth of the encoding networks. It can be concluded that, in the proposed method, the deeper encoding network can learn better representation with rich semantic information. This result is quite different from conventional FSL, where the common perception is that very deep networks cannot be adequately trained under few-shot training conditions, and thus can easily fall into overfitting, resulting in degraded accuracy. We believe the reason about this is that the first phase of our method is actually large-sample training, rather than small-sample task training as in conventional FSLs. And according to the general conclusion of large-sample training, deeper networks tend to learn better performance.

### 3.7 Recognizing potato leaf diseases in natural scenes

We explore the effectiveness of SC-FSL in the task of recognizing plant leaf diseases in natural scenes, a setting with more complex background and lighting variations. Here, the test dataset is PDD collected by us. We follow the data construction scheme of Scenario B, with PlantVillage as the source domain and PDD as the target domain. In the first phase, ResNet 18 and ResNet 50 as encoding networks are trained to learn capacity of extracting feature from the images in PlantVillage, respectively. In the second phase, the few-shot learning sampling mode is adopted on PDD, and five modes are evaluated in the form of 5-way M-shot, where M value is 1,5,10,20 and 30 respectively. The experimental results are shown in **Table 5**.

**Table 5:** Results of potato disease recognition.

M-shot	Accuracy (%)	
	Resnet18	Resnet50
30-shot	69.31 ± 0.50	79.51 ± 0.39
20-shot	68.07 ± 0.51	77.16 ± 0.43
10-shot	64.87 ± 0.52	73.31 ± 0.46
5-shot	60.48 ± 0.54	68.29 ± 0.53
1-shot	43.70 ± 0.63	49.12 ± 0.73

From **Table 5**, for ResNet 18 and ResNet 50, the recognition accuracy increases from 43.7% to 69.31%, and 49.12% to 79.51% when the training samples are increased from 1 to 30, respectively. The number of training samples has an apparent impact on the recognition accuracy. Thus, in order to obtain better performance, as many training samples as possible should be used, even under few samples condition. In addition, with the same number of training samples, the recognition effect of ResNet 50 is significantly higher than that of ResNet 18. In practice, deeper networks should be used as feature extractors.

**Figure 10:**
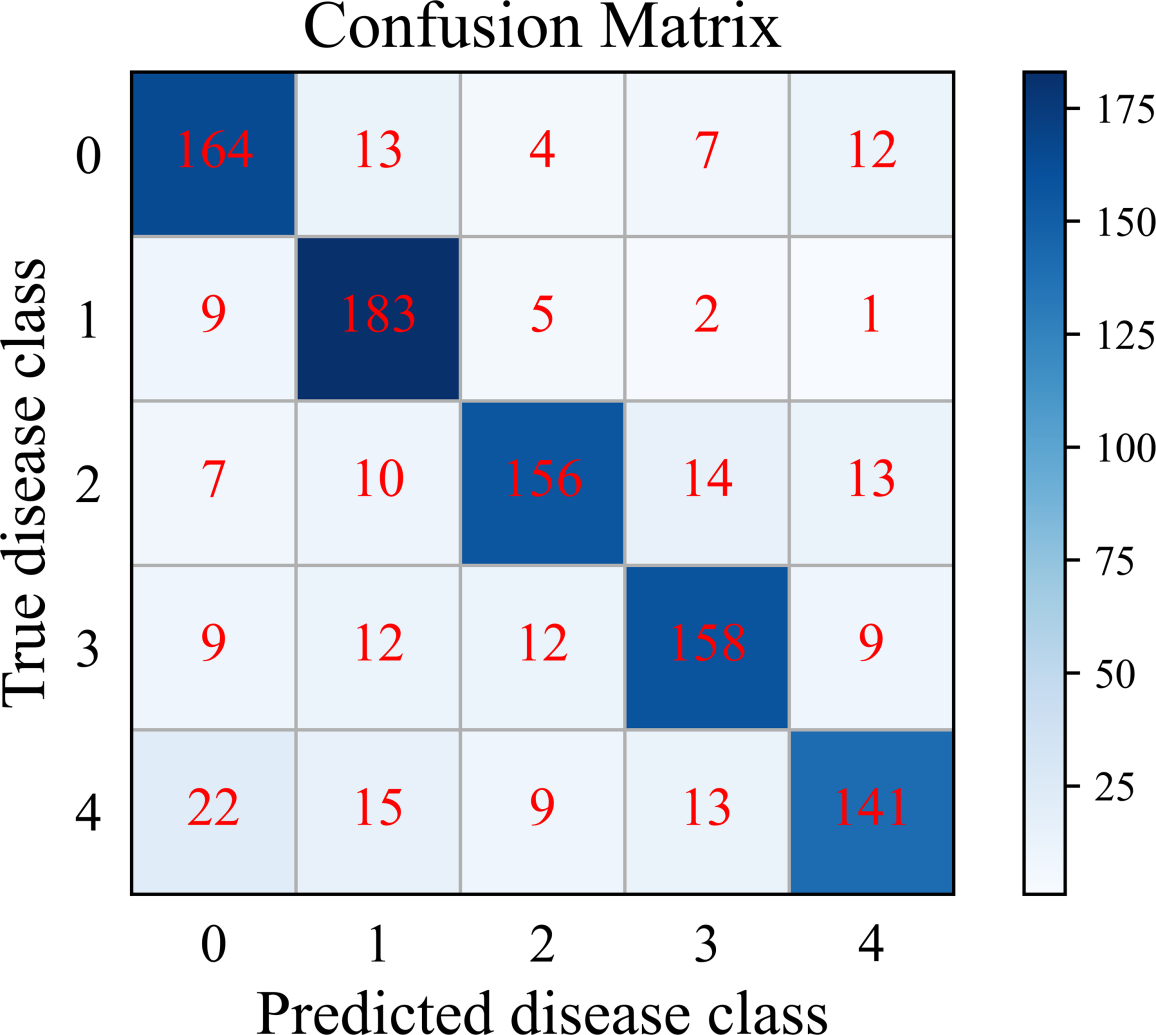
Cumulative confusion matrix of SC-FSL.

We further analyze the classification performance of SC-FSL for each disease class in PDD. When the number of training sample is 30-shot, the cumulative confusion matrix of SC-FSL of 20 episodes is shown in **Figure 10**. In this figure, the potato disease types are denoted as follows: 0 healthy, 1 early blight, 2 late blight, 3 leaf curl, and 4 anthracnose.

As can be seen from **Figure 10**, SC-FSL has the highest recognition accuracy of 91.5% for potato early blight, followed by 82% for healthy leaves. The model has the lowest recognition accuracy of 70.5% for anthracnose. The confusion matrix shows that SC-FSL is most likely to misclassify anthracnose as healthy leaves and early blight. Looking at the potato anthracnose images in PDD, we notice brown circular spots with lighter color in the lesions of a leaf. Some images of early blight also showed circular spots with lighter colors. Therefore, for these images with very similar disease characteristics, the model can easily confuse them, leading to misidentification of anthracnose as early blight. In addition, some of the anthracnose images have a small number of spots, which the model tends to confuse with healthy leaves.

The above results show that our proposed model can also achieve good results in the PDD recognition task under different data distribution conditions in the target domain and source domain.

## 4 Conclusion

Learning through a large number of samples is the key to the success of popular disease recognition based on deep learning. However, in agricultural production, the time and place of disease occurrence are random, which makes it difficult to collect large-scale disease samples. How to obtain a high performance of disease recognition under the condition of only few-shot samples is an open question. At present, the main paradigm is to train an initial model by few-shot samples but multiple tasks in the pre-training stage, and then fine-tune the model with few-shot samples on the specific recognition task. We notice that there are a lot of disease samples that can be used in the pre-training phase, although they do not belong to the classes of the specific task. However, they should share some similar characteristics with the diseases of the specific task. In view of the good generalization performance of contrastive learning, we propose a new few-shot disease recognition paradigm called SC-FSL, that is, big data and contrastive learning in the pre-training stage is used in pre-training phase, and few-shot learning is used in the specific disease recognition stage. Specially, supervised contrastive learning is introduced into the pre-training phase, which allows the utilization of the other disease category information in the training process. It can extend the number of positive samples and force the encoding network to bring samples of the same category closer together in the feature space, which helps to improve the classification of small samples at the later phase. An additional benefit of SC-FSL is the ability to reduce the size of the batch at training time, thus greatly reducing the requirement for training hardware. We conduct a number of experiments to evaluate the factors affecting the effectiveness of the proposed method. Experimental results indicate that the appropriate combination of data augmentations is crucial for learning good representations. The combination of random length-width ratio clipping, random horizontal flipping and color distortion is the best for plant disease recognition. For ResNet 18 as feature extractor, the accuracy of 5-way 1-shot and 5-way 5-shot is 78.55% and 92.90% on PlantVillage, respectively. Label information helps the training of contrastive learning to achieve good learning results with only a small batch size, e.g., for ResNet 18, the optimal batch size is 192, which is much smaller than the batch size for normal contrastive learning methods. In comparative experiments, the recognition accuracy of SC-FSL over PlantVillage outperforms the other nine FSL algorithms in all scenarios. Furthermore, the encoder learned on PlantVillage still achieves high accuracy as a feature extractor on PDD that is completely different from PlantVillage, without any fine-tuning. This result indicates that SC-FSL has good generalization performance in cross-domain recognition tasks. We believe that this high performance essentially comes from the fact that the encoder is learnt through large samples in the contrastive learning process. When the encoder acts as a feature extractor in the few-shot recognition task, it has a good clustering effect on samples of the same category in the feature space, which improves recognition accuracy of the disease.

## Data availability statement

The original contributions presented in the study are included in the article/Supplementary Material. Further inquiries can be directed to the corresponding authors.

## Author contributions

JM: Conceptualization, Formal analysis, Investigation, Software, Writing – original draft. QF: Conceptualization, Formal analysis, Methodology, Software, Writing – original draft, Writing – review & editing. JY: Data curation, Formal analysis, Investigation, Software, Writing – review & editing. JZ: Conceptualization, Methodology, Writing – review & editing. SY: Conceptualization, Formal analysis, Investigation, Validation, Writing – review & editing.
